# Italian Association of Clinical Endocrinologists (AME) position statement: a stepwise clinical approach to the diagnosis of gastroenteropancreatic neuroendocrine neoplasms

**DOI:** 10.1007/s40618-014-0119-0

**Published:** 2014-07-20

**Authors:** Franco Grimaldi, Nicola Fazio, Roberto Attanasio, Andrea Frasoldati, Enrico Papini, Francesco Angelini, Roberto Baldelli, Debora Berretti, Sara Bianchetti, Giancarlo Bizzarri, Marco Caputo, Roberto Castello, Nadia Cremonini, Anna Crescenzi, Maria Vittoria Davì, Angela Valentina D’Elia, Antongiulio Faggiano, Stefano Pizzolitto, Annibale Versari, Michele Zini, Guido Rindi, Kjell Öberg

**Affiliations:** 1 Endocrinology and Metabolic Disease Unit, Azienda Ospedaliero-Universitaria “S. Maria della Misericordia”, P.le S.M. della Misericordia, 15-33100, Udine, Italy; 2 Unit of Gastrointestinal and Neuroendocrine Tumors, European Institute of Oncology, Milan, Italy; 3 Endocrinology Service, Galeazzi Institute IRCCS, Milan, Italy; 4 Endocrinology Unit, Arcispedale S. Maria Nuova IRCCS, Reggio Emilia, Italy; 5 Endocrinology Unit, Regina Apostolorum Hospital, Albano Laziale, Rome, Italy; 6 Oncology and Hematology Unit, Regina Apostolorum Hospital, Albano Laziale, Rome, Italy; 7 Endocrinology Section, Regina Elena National Cancer Institute, Rome, Italy; 8 Gastroenterology Unit, Azienda Ospedaliero-Universitaria “S. Maria della Misericordia”, Udine, Italy; 9 Diagnostic Imaging Unit, Regina Apostolorum Hospital, Albano Laziale, Rome, Italy; 10 Dipartimento Servizi di Diagnosi e Cura, AUSL 22 Regione Veneto, Bussolengo, VR Italy; 11 Medicina Interna ad indirizzo Endocrinologico, Azienda Ospedaliera Universitaria Integrata, Verona, Italy; 12 Endocrinology Unit, Maggiore and Bellaria Hospital, Bologna, Italy; 13 Pathology Unit, Regina Apostolorum Hospital, Albano Laziale, Rome, Italy; 14 Medicina Interna D, Azienda Ospedaliera Universitaria Integrata, Verona, Italy; 15 Genetic Service, Azienda Ospedaliero-Universitaria “S. Maria della Misericordia”, Udine, Italy; 16 Department of Clinical Medicine and Surgery, Federico II University, Naples, Italy; 17 Pathology Unit, Azienda Ospedaliero-Universitaria “S. Maria della Misericordia”, Udine, Italy; 18 Nuclear Medicine Service, Arcispedale S. Maria Nuova IRCCS, Reggio Emilia, Italy; 19 Department of Endocrine Oncology, University Hospital, Uppsala, Sweden; 20 Institute of Pathology, Policlinico A. Gemelli, Università Cattolica del Sacro Cuore, Rome, Italy

**Keywords:** Neuroendocrine tumors, Diagnostic work-up, Markers, Imaging, Incidental findings, Non-functioning tumors, Carcinoid syndrome, Gastrinoma, Insulinoma, NET, NEC, NEN

## Introduction

### Why this document

Neuroendocrine neoplasms (NENs) can arise almost throughout the entire body and share common morphological, ultrastructural, and immunohistochemical characteristics.

Neuroendocrine neoplasms are an emerging entity that can occur at any age, with the median age at diagnosis in the late fifth decade and an age-related incidence increase. About two-thirds involve the gastro-entero-pancreatic (GEP) tract and epidemiological studies show their increasing incidence [1]. In the last decades, the overall reported incidence of GEP-NENs increased from 1.0 to 5.25/100.000 persons/year, with a present estimated prevalence of 35/100.000 [1–10]. Physicians’ awareness, endoscopic screening and increased sensitivity of diagnostic tools may at least in part explain this growing trend.

Most guidelines are focused on staging, treatment and follow-up of NENs. However, an appropriate clinical suspicion and a correct diagnostic work-up are critical starting points. A multidisciplinary approach, moreover, is crucial to provide a timely and integrated care. Hence, this document is neither a review, nor a guideline; rather, it is a clinical guide for a stepwise and integrated diagnostic work-up of GEP-NENs. Hopefully, this will result in a correct utilization of resources and optimization of the cost/benefit ratio.

### Methodology

The grading of recommendations, assessment, development, and evaluation (GRADE) system was adopted for the present position statement [11–14]. Briefly, the GRADE system classifies evidence into four quality levels (high, moderate, low, or very low), and recommendations into two grades (strong or weak).

Whenever possible, the level of evidence (LoE) has been ranked as follows: very low (⊗○○○), low (⊗⊗○○), moderate (⊗⊗⊗○), and high (⊗⊗⊗⊗). “Very low quality” evidence corresponds to unsystematic clinical observations (case report, case series) or indirect evidence (e.g., surrogate end points); “low quality” evidence corresponds to observational studies or randomized controlled trials (RCT) with major limits; “moderate quality evidence” corresponds to RCTs with limitations or rigorous observational studies; and “high quality evidence” corresponds to well performed RCTs and strong evidence from unbiased observational studies [13].

We labeled as “recommendations” and “suggestions” the strong and weak recommendations, respectively. Each recommendation/suggestion is based on the quality of supporting evidence, downgraded or upgraded according to adjunctive factors (e.g., inconsistency of results, indirectness of evidence, lack of precision and limited number of relevant publications downgrade the recommendation/suggestion; large effect size, narrow confidence intervals, clinically very significant end points upgrade the recommendation/suggestion), and the level of panel agreement [13].

### Definitions

Neuroendocrine neoplasms neoplastic cells possess features of both neural and epithelial cells. Therefore, in line with the WHO classification, the term neuroendocrine will be adopted throughout this document [15].

WHO recommends the use of the term “neuroendocrine neoplasm” (NEN) to indicate low- to high-grade lesions. The term “neuroendocrine tumor” (NET) will be used throughout this document, due to its widespread diffusion, to indicate low- to intermediate-grade lesions and the term “neuroendocrine carcinoma” (NEC) to indicate high-grade lesions. Terms like “carcinoids” and the embryological classification of GEP-NENs in tumors of foregut (thymus, esophagus, lung, stomach, duodenum, pancreas), midgut (appendix, ileum, cecum, ascending colon) and hindgut (distal colon and rectum) will be avoided.

### Classification

In the last 10 years WHO has repeatedly revised the pathologic classification of GEP-NENs (Table 1) [16].

**Table 1:** WHO classifications of GEP-NENs

WHO 1980	WHO 2000	WHO 2010
I. Carcinoid	Well-differentiated endocrine tumor Well-differentiated endocrine carcinoma Poorly differentiated endocrine carcinoma/small-cell carcinoma	Neuroendocrine tumors NET G1 (Grade 1) NET G2 (Grade 2) Neuroendocrine carcinoma NEC G3 (Grade 3): Large-cell NEC small-cell NEC
II. Mucocarcinoid III. Mixed carcinoid-adenocarcinoma forms	Mixed exocrine–endocrine carcinoma	Mixed adeno-neuroendocrine carcinoma (MANEC)
IV. Pseudotumor lesions	Tumor-like lesions	Hyperplastic and preneoplastic lesions

According to the 2010 classification, **NET G1** includes the “carcinoids” or “well-differentiated tumors” of the 1980 and 2000 WHO classifications. These tumors are usually indolent, but can occasionally behave as malignant.

**NET G2** may be considered a “grey zone”, with heterogeneous behavior, and requires a tailored management.

**NEC (G3)** is a malignant neoplasm with an aggressive clinical course.

**MANEC** has a malignant phenotype with features of both adenocarcinoma and NET. This definition requires the presence of at least 25 % of each component. Neuroendocrine cells are usually interspersed and the two populations may be identified only by immunohistochemistry (IHC). Less frequently, neuroendocrine cells may be grouped in distinct regions that are recognized by light microscopy.

The WHO 2010 classification strongly relies on tumor grading. Grading relates to the biological aggressiveness of the neoplasm, whereas differentiation indicates its similarity to the tissue of origin [15]. The clinical behavior of NENs may be basically predicted by their grading, staging, and evidence of hormonal syndromes. All these data should be collected and weighted to establish the prognosis and management of the patient.

#### Grading assessment

The grade of a tumor is the primary predictor of its clinical outcome. Grading is based on the proliferation rate of the tumor, as assessed by the Ki-67 cell labeling and by the mitotic count (number of mitosis × 10 high power fields—HPF) (Table 2) [15–23].

**Table 2:** Grading system for GEP-NENs (adapted from 19)

	Ki-67 index (%)^a^	Mitotic count/10 HPF^b^
NET G1	≤2	<2
NET G2	3–20	2–20
NEC G3	>20	>20

^a^Assessed by MIB-1 labeling in at least 2,000 tumor cells in high nuclear density (“hot spot”) areas

^b^10 HPF = 2 mm^2^, at least 50 optical fields in high-density mitotic areas

Visual estimates are currently used as the standard technique for evaluating both Ki-67 and the mitotic count [24, 25]. Several areas should be assessed within the tumor to reduce the risk of evaluation bias due to intratumoral heterogeneity. Densely stained regions (“hot spots”) should be preferentially evaluated. Results from these areas should be reported as a single percentage reflecting the highest identified count [16, 21, 22].

Potential pitfalls and limitations are:

- technical problems (e.g., tissue processing, differences in Ki-67 antibodies, etc.);
- intratumoral heterogeneity and sampling limitations (e.g., a single biopsy sample may not be representative of the tumor grade within the whole neoplastic mass) [24, 26];
- discordances:
  I. between the proliferative rate and the degree of differentiation (e.g., a morphologically well-differentiated NEN may exhibit a high proliferative rate);
  II. between the predictive value for prognosis and that for treatment response (e.g., Ki-67 is a reliable predictor of disease progression and overall survival (OS), but seems a less efficient predictor of response to medical treatment) [27].

#### Pathologic staging

**Fig. 1:**
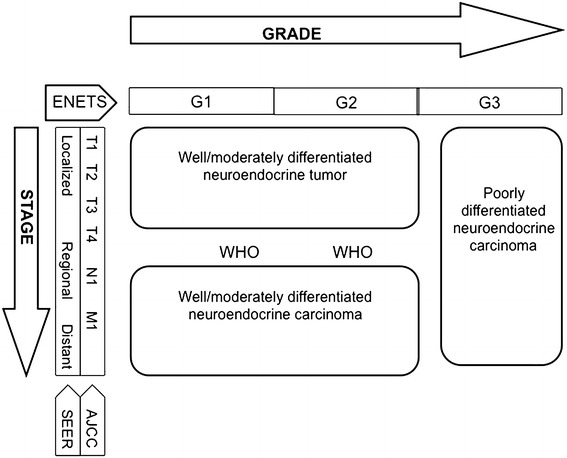
Integrated pathologic and biologic classification (*modified from 15*)

GEP-NENs are staged according to tumor size, site of origin, and locoregional or distant spreading [21–23]. The staging information is integrated with the 2010 WHO classification to stratify the prognostic risk and optimize the therapeutic and follow-up strategies (Fig. 1). 
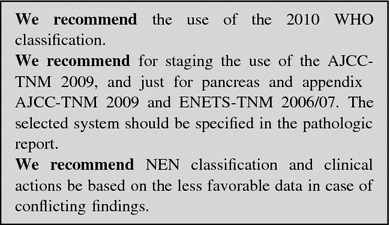

## Diagnostic tools

### Histology, cytology, immunohistochemistry, and molecular biology

#### Morphologic criteria

Pathologic assessment is required for the diagnosis, classification and staging of NENs.

GEP-NENs present a broad architectural spectrum [28]. Well-differentiated tumors show an organoid pattern that ranges from solid nests to micro–macrotrabecular/gyriform pattern. A rich sinusoidal vascularity is usually observed. Stromal fibrosis, amyloid deposition, and calcification may be present. Necrosis can be present either as large infarct-like areas or as punctate foci in the center of neoplastic nests. Regardless of their growth pattern, NEN cells have a similar cytological appearance: small- to medium-size cells with round to oval shape and eosinophilic, lightly granular, cytoplasm. The nuclei are usually centrally placed, fairly uniform, with a finely dispersed (“salt and pepper”) chromatin pattern. Rarely, the neoplastic cells have a “plasmocytoid appearance” due to peripherally located nuclei. Nucleoli are usually inconspicuous or absent. Intracytoplasmatic hyaline globules or nuclear pseudoinclusions may be seen.

High-grade NENs are composed of small or large-to-intermediate cells with high-grade features (marked nuclear atypia, multifocal necrosis, high mitotic index) and diffuse growth, sometimes with organoid feature resembling NEN.

The subgroup of GEP-NENs with Ki67 >20 % (and therefore G3 according to WHO 2010), but with a morphology of well-/moderately differentiated tumor should be considered low/intermediate rather than high-grade NENs [29].

Cytological specimens, which may be the only source of diagnostic material, pose some problems for clinical management. Cytology effectively separates high-grade NENs from low-grade NENs, but the distinction between low- and intermediate-grade NENs may be impossible. The diagnostic accuracy of aspiration techniques may be limited by the small sample size, the suboptimal reproducibility and the risk of contamination from contiguous tissues.

Cytology is gaining a major role for the diagnosis in duodeno-pancreatic tumors. The endoscopic ultrasonography (EUS) fine needle aspiration (FNA) technique appears reliable, with a reported specificity of about 75 %, sensitivity of 87.5 %, accuracy of 89 %, positive predictive value (PPV) of 93 %, and negative predictive value (NPV) of 60 % [30–32].

#### Immunohistochemistry and molecular biology techniques

*Neuroendocrine differentiation* Synaptophysin (a small vesicle-associated marker) and Chromogranin A (CgA, a large secretory granule-associated marker) are useful IHC markers for the diagnosis of NENs. In NEC, the staining for both these markers is required to confirm the diagnosis, because CgA may be negative [15]. Routine IHC staining for peptide hormones and bioamines is not recommended. Other neuroendocrine markers, such as PGP.9.5, NSE, CD56, NSP-55, are of questionable specificity and clinical usefulness.

*Prognostic markers* proposed in addition to Ki-67 are CK19, CD117, CD99, p53, Her/2, CEACAM1, E-cadherin, β-catenin, hHAS-1, FGF13, PLGF, PAX-8, PTEN. None is presently recommended for clinical practice. The research of circulating tumor cells or the use of microRNAs is not indicated for routine use [33, 34].

*Markers of primary site* These markers may be a key for determining the unknown primary tumor in metastatic lesions. The most useful are [35]:

- TTF-1, indicative of pulmonary or thyroid origin;
- serotonin and CDX-2, indicative of intestinal origin;
- PAX-8 and histidine-decarboxylase, indicative of pancreatic origin;
- xenin, indicative of duodenal origin.

*Markers predictive of response to specific treatments* These biomarkers are not indicated for routine diagnostic practice. They include [19]:

- somatostatin receptors (SSTR)-2A (IHC determination at the cell membrane level), for planning the treatment with somatostatin analogs (SA);
- Akt/mTOR pathway molecules (PIK3, PTEN, TSC2), for treatment with everolimus;
- thymidylate synthase, for treatment with antifolates;
- ERCC-1, for treatment with platinum;
- topoisomerase Iiα, for treatment with etoposide;
- epigenetic events, as methylation of MGMT promoter, for treatment with alkylating agents.

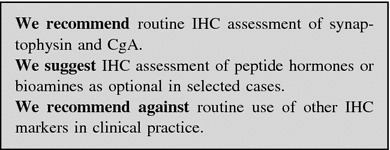


#### Working with the pathologist and his pathologic report

The modality and timing of sampling techniques should be planned by a multidisciplinary team.

The pathologist should be provided with accurate clinical information including signs and symptoms, laboratory findings and imaging studies [36].

The ideal pathologic report should include:

- description of the macroscopic specimen;
- tumor size (three dimensions);
- description of cell features and histologic architecture;
- differentiation (well or poorly differentiated);
- IHC findings (CgA and synaptophysin routinely, SSTR2A when appropriate (e.g., when functional imaging for SSTR2 is negative);
- Ki-67 and mitotic count;
- completeness of resection, distance of the surgical margins from the tumoral edge, depth of invasion;
- signs of malignancy (angiolymphatic and/or perineural invasion, necrosis, infiltration of the capsule and/or of gastrointestinal (GI) wall and/or surrounding tissues);
- number of examined lymph nodes, and number of lymph node metastases; presence of micrometastases; diameter of largest metastasis;
- presence of distant metastases, if demonstrated;
- functional activity (if appropriate).

The report should be concluded with the WHO diagnosis and classification of the lesion (NET G1–G2 or NEC G3) based on proliferative index (Ki-67 and/or mitotic count), and with the tumor stage (the staging system should be specified).

The minimum pathology data set for resected specimens (both primary and metastatic) should include [37]:

- site;
- diagnosis (e.g., pure neuroendocrine neoplasm);
- differentiation (i.e., well or poor);
- proliferation (i.e., G1 or G2 or G3).

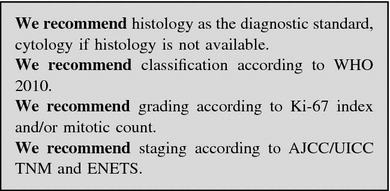


#### Genetic assessment

Approximately 5–10 % of GEP-NENs have a hereditary background as part of tumor susceptibility syndromes: multiple endocrine neoplasia type 1 (MEN-1), von Hippel-Lindau disease (VHL), neurofibromatosis type 1 (von Recklinghausen disease, NF1) and the tuberous sclerosis complex (TSC). All are inherited autosomal dominant disorders [38].

*MEN*-*1* GEP-NENs are the second most common manifestation of MEN-1, reported in 30–70 % of cases in different series [mostly non-functioning (NF)] [39, 40]. A germ-line *MEN*-*1* mutation is identifiable in about 80–90 % of familial cases [41] and in about 42 % of sporadic cases [42]. Germline mutations arise de novo without any family history in approximately 10 % of patients [43]. *MEN*-*1* mutation testing should be offered to index cases and to their first-degree relatives, even if asymptomatic [40]. Genetic counseling is recommended [40]. The family members who carry the *MEN*-*1* mutation require routine surveillance for early detection of endocrine tumors, whereas those who do not carry the mutation can be reassured. When molecular genetic testing is not available locally, patients highly suspected for MEN-1 should be addressed to a referral centers. No genotype/phenotype correlations have been demonstrated in MEN-1 syndrome [44, 45].

*VHL* Endocrine pancreatic NF tumors occur in 11–17 % of patients with VHL disease [46]. The penetrance of *VHL* mutations is almost complete by age 65 years [47]. Genetic testing detects mutations in virtually all affected individuals [48] and should be offered to all individuals with clinical evidence of VHL and to first-degree relatives. As ophthalmologic screening for those at risk for VHL disease begins before age five, molecular genetic testing is suggested also in young asymptomatic children [49, 50].

*NF1* GEP-NENs occur in 1 % of the NF1 patients [51]. Half of affected individuals have NF1 as the result of a de novo mutation. The offspring of an affected individual is at a 50 % risk of inheriting the altered *NF1* gene, and the disease manifestations are extremely variable, even within the same family [52]. Molecular testing for NF1 is not usually recommended in the clinical practice: screening for NF1 mutations is useful only in individuals who do not completely fulfill the NIH diagnostic criteria.

*TSC* A few cases of pancreatic (p)NENs have been described in patients with TSC [53–55]. The diagnosis of TSC is usually based on clinical findings and mutations can be identified in approximately 85 % of individuals who meet the diagnostic criteria [56]. Two-thirds of affected individuals have TSC as the result of a de novo mutation.
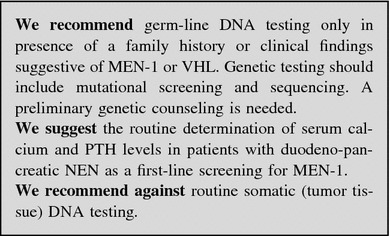


### Laboratory assessment

The determination of GEP-NENs serum markers should not be used as a first-line diagnostic tool whereas it is appropriate for monitoring the response to treatment and for long-term follow-up [57, 58].

Serum markers should be determined after:

1. an established diagnosis or strong clinical suspicion of GEP-NEN;
2. exclusion of physiologic and pathologic confounding conditions.

NEN markers may be regarded as “unspecific” or “disease-specific”.

#### “Unspecific markers”

##### Chromogranin A

Chromogranin A is a widely employed serum marker for GEP-NENs, but its use presents limitations [59]. CgA circulates under different antigenic forms and no universal calibration standard is available [60]. IRMA and RIA results may be considered roughly equivalent [61], but the reference intervals are variable and results obtained with different assays cannot be compared.

Chromogranin A level may be increased in a number of pathologic conditions (Table 3), and in healthy subjects after eating or physical exercise. Accordingly, CgA levels are highly variable in the general population [62], and may partially overlap between GEP-NEN patients and controls. Hence, CgA has a poor first-line diagnostic value [5, 60, 62–66].

**Table 3:** Potential confounders causing CgA increase [64]

Neoplastic (other than GEP-NENs)
Breast cancer Prostate cancer Ovarian cancer Hepatocarcinoma
Pancreas adenocarcinoma Colon cancer
Non-neoplastic
Kidney or heart failure Endocrine diseases (hyperthyroidism, hyperparathyroidism) Local or systemic inflammatory disease Chronic obstructive broncho-pulmonary disease Gastro-enteric pathologies: chronic atrophic gastritis, pancreatitis, inflammatory bowel disease, cirrhosis, chronic hepatitis

Proton pump inhibitors (PPIs) increase (up to sevenfold) CgA levels. The effects of PPIs persist for several days after drug discontinuation. Therefore, CgA testing should be performed after an at least 2-week PPIs withdrawal [62, 67]. The effects of H2-receptor antagonists (H2RAs) on CgA are still controversial [68].

Diagnostic accuracy of CgA depends on different variables:

- tumor burden (sensitivity 60–100 vs. 29–50 % in metastatic and localized disease, respectively) [62, 64, 69];
- type and site of tumor (sensitivity 96 vs. 75 % in functioning and NF tumors, respectively) [63, 70].

##### Other unspecific markers

Neuron-specific enolase (NSE) is an enzyme found in neuroectoderm-derived cells. The presence of NSE has been reported in thyroid and prostate carcinomas, neuroblastomas, small-cell lung carcinomas, and pheochromocytomas. The clinical usefulness of this marker is hampered by its poor specificity [71]. NSE level is elevated in 30–50 % of patients with NEN, particularly those with poor differentiation. The combined determination of NSE and CgA may improve sensitivity in GEP-NEN diagnosis [72].

Pancreatic polypeptide (PP) is secreted by specialized pancreatic islet cells and inhibits gut motility and pancreatic exocrine secretion. PP has been proposed for the diagnosis and monitoring of NF pNENs, as its combination with CgA increases sensitivity up to 93 % [69]. Its routine use is not recommended due to the low diagnostic performance (sensitivity 63 % and specificity 81 %). PP levels may increase in old age, diarrhea, laxative abuse, gut inflammatory processes and chronic renal disease.

Beta subunit of human chorionic gonadotropin (hCG), a glycoprotein synthesized by the syncytiotrophoblastic cells of the placenta during pregnancy, may be increased in patients with pNENs [73], but has no use in every day practice.

As a whole, the clinical usefulness of the above reported markers is limited.

#### “Specific markers”

##### 5-HIAA

5-HIAA, the main urinary metabolite of human serotonin, is determined by HPLC on 24 h urine samples. Results may be expressed as absolute values or as a ratio to creatinine excretion.

Some pre-analytical variables, mostly tryptophan/serotonin-rich foods and drugs, may interfere with serotonin metabolism (Table 4) [74]. These products should be avoided prior to urine collection, respectively, for at least 72 and 24 h [64].

**Table 4:** Drugs and foods interfering with 5-HIAA assay

False negative results
Acetylsalicylic acid Phenothiazines: chlorpromazine, promethazine Imipramine and MAO-inhibitors ACTH Ethanol MethylDOPA and hydrazine derivatives Ketoacids LevoDOPA Isoniazid, methenamine, gentisic and homogentisic acid Streptozotocin Heparin
False positive results
Acetaminophen, naproxen, phenacetin Caffeine, nicotine Coumaric acid Diazepam Ephedrine Fluorouracil, melphalan Phenobarbital Phentolamine, reserpine, guaifenesin, mephenesin Methamphetamine, Phenmetrazine Methocarbamol Mesalamine Foods: bananas, avocados, kiwi, pineapples, peanuts, tomatoes, plums, eggplants, walnuts, pecans, coffee, tea, cocoa/chocolate, vanilla, sweets, and cookies (sugar and marmalade are allowed)

The normal 5-HIAA urinary excretion ranges from 2 to 8 mg/day, but unspecific elevations (up to 30 mg/day) may be found in malabsorption syndromes, such as celiac and Whipple’s disease [75–77].

The determination of 24-h urinary excretion of 5HIAA has a sensitivity of over 90 % and a specificity of 90 % for full-blown carcinoid syndrome (CS, see “Box 2, Carcinoid syndrome”). Urinary 5-HIAA excretion in these patients is reportedly higher than 90 mg/day (up to 2,000 mg/day). The test sensitivity, however, is definitely lower in absence of clinical symptoms [77, 78].

There is a possibility to analyze plasma 5-HIAA which might replace urinary-5-HIAA in a future [79].

Various blood serotonin assays have been proposed, but their actual accuracy has not been established. False positives may occur due to several interfering factors, as the release of platelet serotonin or the previous ingestion of tryptophan/serotonin-rich foods [80]. Accordingly, serotonin determination is not recommended in clinical practice.

##### Gastrin

Gastrin determination has a key role in the evaluation of patients with signs and symptoms suggestive of Zollinger–Ellison syndrome (ZES, see “Box 3, Gastrinoma”).

Various antigenic isoforms of gastrin circulate in the blood. Care must be taken because some commercial immunoassay kits detect only the gastrin-17 molecule [81] and may cause false positive results.

Hypergastrinemia is commonly defined as a fasting serum gastrin above 100 pg/mL. Simultaneous measurement of gastric pH on a single sample is needed to rule out secondary hypergastrinemia due to other causes. In achlorhydria, pernicious anemia or atrophic gastritis high gastrin levels are usually associated to high (i.e., >4) pH values. On the contrary, serum gastrin levels >1,000 pg/mL combined with a <2 gastric pH are virtually diagnostic of ZES. Falsely elevated gastrin levels may be due to a few drugs (Table 5) that should be discontinued at least 2 weeks before the test [77, 82–86].

**Table 5:** Main drugs and foods that may interfere in gastrin assay

False negative results
Acetylsalicylic acid LevoDOPA
False positive results
Hypochlorhydria/achlorhydria due to chronic use of PPIs and H2RAs or chronic atrophic gastritis (often associated with pernicious anemia) *Helicobacter pylori* infection Gastric outlet obstruction Renal failure Antral G-cell syndromes Short-bowel syndrome Retained antrum

In general, gastrin levels are higher in pancreatic than in duodenal NENs, and are proportional to tumor burden and in patients with metastatic disease, exceedingly high gastrin levels may be observed. However, the majority of patients with ZES show mildly elevated (e.g., 150–1,000 pg/mL) gastrin levels, partially overlapping those of patients with renal insufficiency, small-bowel resection, retained gastric antrum, or on potent antisecretory drugs [87]. When the diagnosis is equivocal, a secretin stimulation test is needed. A gastrin increase >120 pg/mL over basal level is considered diagnostic [88].

##### Insulin

The occurrence of repeated symptomatic hypoglycemia (<60 mg/dL) is suspicious for insulinoma (see “Box 4”) in subjects without diabetes. The diagnosis is confirmed by the presence of non-suppressed insulin levels in presence of low glucose levels (see “Spontaneous hypoglycemia”). To rule out a spurious hypoglycemia, laboratory processing should not be delayed. In subjects with leucocytosis glucose determination should be repeated with a collection tube that contains an inhibitor of glycolysis.

In presence of an episode of spontaneous severe hypoglycemia with hyperinsulinism, the simultaneous measurement of serum C-peptide and beta-hydroxybutyrate is appropriate. If factitious hypoglycemia is suspected, urinary sulfonylureas should be tested as well. The work-up may be completed with the measurement of serum proinsulin [89]. This test, even if not widely available, is diagnostic of insulinomas secreting immature forms of insulin.

In selected patients with endogenous hyperinsulinism, autoimmune hypoglycemia, suspected on the basis of negative imaging tests and coexistence of autoimmune disorders, should be ruled out with the determination of insulin autoantibodies [90].

If the patient is not hypoglycemic when observed, the association of severe hypoglycemia with non-suppressed insulin levels should be seeked under the conditions in which hypoglycemia would be expected (see provocative testing, “Spontaneous hypoglycemia”) [89].

##### Other specific markers

Glucagon: Glucagonoma is associated with serum glucagon concentrations higher than 500 pg/mL and a characteristic clinical syndrome (diabetes mellitus and cutaneous manifestations, such as migratory necrolytic erythema, nail dystrophies, stomatitis, etc.) [91]. Glucagon concentrations higher than 1,000 pg/mL are virtually diagnostic for the disease, but some patients may exhibit levels within the physiologically elevated range. Moderate elevations in serum glucagon may be caused by protracted fasting in normal subjects or by renal and hepatic failure, trauma, sepsis, pancreatitis, abdominal surgery, and Cushing’s syndrome.

Due to the fast degradation of glucagon in vitro, blood must be collected in test tubes containing aprotinin and should be rapidly delivered to the laboratory. Results obtained with different glucagon assays may profoundly differ, due to the different calibration standards and the variable cross-reactivity with glucagon isoforms.

Vasointestinal peptide (VIP): Vasointestinal peptide-secreting tumors cause the Verner–Morrison syndrome, characterized by variable combination of watery diarrhea (>700 mL/day even during fasting, with tea-colored, odorless stools), hypokalemia, achlorhydria, weight loss, metabolic acidosis, hypercalcemia, glucose intolerance, and flushing. The diagnosis is established by high-volume secretory diarrhea associated with VIP levels higher than 75 pg/mL (to be confirmed by a second RIA determination) [92, 93]. VIP blood concentration is, in fact, extremely low in healthy subjects. Commercial kits are available, but their use is usually limited to tertiary referral centers.
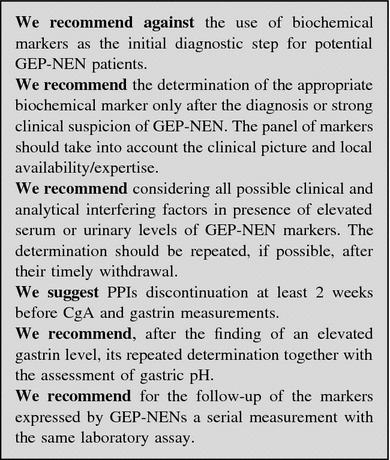


### Imaging procedures

#### Radiologic procedures

##### Ultrasonography

Transabdominal US is an inexpensive, safe, rapid and non-invasive tool. US accuracy is, however, operator dependent and its sensitivity is generally low (13–27 %), when compared with MultiDetector CT (MDCT) and magnetic resonance imaging (MRI) [94]. In case of pNEN, a mean 39 % US detection rate has been reported [95, 96].

Contrast-enhanced US (CEUS) enables identification of hypervascular lesions, even in case of fast-flow tumor circulation, as in NF pNENs. Therefore, CEUS is significantly superior to B-mode US both in the detection of NF pNENs and in the diagnosis of liver metastases, visualized as hyperenhancing non-homogeneous lesions [96–98], with a reported sensitivity of 82 % [99, 100]. US may help in defining complications of advanced disease (i.e., biliary stricture) and/or guide diagnostic or therapeutic procedures [101].

Endoscopic ultrasonography and EUS-guided FNA, a fundamental procedure for the diagnosis of pNENs [96, 102, 103], will be treated in “Pancreatic NENs”.

##### Multislice triple phase CT

Multidetector CT is considered the first choice imaging modality for detection, staging and follow-up of GEP-NENs. When compared to conventional CT, MDCT allows a markedly higher spatial and temporal resolution. MDCT sensitivity and specificity are increased due to multiphase scanning. Images should be acquired in precontrast, arterial, portal and equilibrium phases.

Non-functioning pNENs and NEN liver metastases typically appear as hypervascular lesions. In the evaluation of NF pNENs, the combination of arterial dominant-phase and portal venous-phase CT improves the detection of primary tumors and hepatic metastases [96].

Reported mean sensitivity and specificity of MDCT are 73 % (63–82 %) and 96 % (83–100 %) for pNENs, and 82 % (78–100 %) and 92 % (83–100 %) for liver metastases, respectively [104–106].

When a small ileum lesion is suspected, MDCT enterography can be performed by distending the small bowel with a large volume of neutral or low-attenuating oral contrast medium [107–109]. The reported sensitivity and specificity of MDCT enterography are variable, ranging from 50 to 85 % and from 25 to 97 %, respectively.

Due to radiation exposure, MDCT examination should be tailored, particularly in young people, to reduce the scanned volume and the number of phases.

##### MRI

Like MDCT, MRI offers a high spatial and time resolution with the possibility of multiplanar acquisition and reconstruction and multiphase examination after contrast injection. Along with the absence of ionizing radiations, an advantage of MRI over MDCT is the intrinsic signal difference (contrast) between the neoplasm and the healthy parenchyma. This characteristic is increased with imaging sequences based on proton diffusion. If compared with MDCT, the major drawbacks of MRI are the higher cost, lower accessibility and longer scanning time. Furthermore, MRI is more dependent on patient cooperation. At MRI, GEP-NENs show the same enhancement characteristic described for MDCT. As for contrast medium, Gadolinium-based (Gd-EOB DTPA) agents (Primovist for MRI) should not be used in patients with advanced renal function impairment.

Magnetic resonance imaging demonstrates a particular sensitivity for liver, bone, soft-tissue, and central nervous system metastases [87, 95]. Multiphase CT scan and MRI have similar effectiveness in the detection of islet cell tumors if fat-saturated T1-weighted and delayed enhanced T1-weighted sequences are included.

In clinical practice, MRI should be used when MDCT does not offer clear-cut results or when contrast medium is contraindicated [95]. Due to the absence of radiation exposure, MRI is used, in association with US, either as a screening image modality in young patients or in long-term surveillance [110].
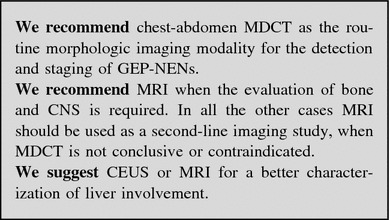


#### Nuclear medicine procedures

##### SSTR functional imaging

Up to 80 % of GEP-NENs express primarily SSTR2 and SSTR5: this feature enables imaging with SA compounds, labeled with radioactive tracers.

The most common radiopharmaceutical SA is ^111^In-pentetreotide (commercially available as Octreoscan^®^) used for scintigraphy, SPECT and SPECT/CT [111, 112]. Modern hybrid acquisition systems as SPECT/CT allow a coregistration of functional and morphologic imaging, which improves the localization of lesions [113].

Due to its high affinity to SSTR2, Octreoscan^®^ shows a higher detection rate of NEN lesions as compared to conventional imaging, with a sensitivity ranging from 67 to near 100 % [114–119].

Among other radiolabeled SA, ^68^Ga-DOTA-D-Phe1-Tyr3-octreotide (DOTATOC) binds SSTR2 and SSTR5 with higher affinity than Octreoscan^®^ [120]. In light of higher spatial resolution (3–5 mm) and better quantification of tracer uptake offered by PET in comparison with scintigraphy, PET and PET/CT scan with ^68^Ga-DOTATOC have significant advantages over SRS imaging, particularly in organs with high physiologic uptake (e.g., liver) and in case of small lesions (<1.5 cm) [121–123]. Furthermore, ^68^Ga-DOTATOC has proven to be superior to CT and bone scintigraphy in the detection of bone metastases from GEP-NENs [124].

Similar results have been obtained with PET imaging using other ^68^Ga-labeled peptides (e.g., ^68^Ga-DOTATATE and ^68^Ga-DOTANOC) [125–130]. PET/CT with ^68^Ga-labeled SA is quite effective, both in terms of diagnostic accuracy and impact on clinical management [131–134]. Accordingly, this imaging procedure is recommended for routine use [73]. PET/CT with ^68^Ga-labeled SA is presently available at a limited number of institutions, but will hopefully become diffusely adopted worldwide in the next future (Table 6).

**Table 6:** Comparison between Octreoscan and Ga-DOTA-peptides

	Availability	Duration	Accuracy	NPV	PPV
^111^In-pentetreotide (Octreoscan^®^)	Widespread	2 days	++	++	+++
^68^Ga-DOTA-conjugate peptides	Low	2 h	+++	+++	+++

Clinical indications for nuclear imaging based on radiolabeled SA are [135]:

- primary tumor localization and staging;
- restaging (detection of residual, recurrent or progressive disease);
- SSTR status evaluation (patients with high positivity are more likely to respond to octreotide therapy);
- response to therapy monitoring;
- selection of patients eligible for peptide receptor radionuclide therapy.

As octreotide therapy can theoretically interfere with ^111^In-pentetreotide uptake, a brief (1–2 months) withdrawal of long-acting SA or a transient switch to short-acting SA should be considered [135].

##### PET with other tracers

^18^F-FDG-PET/CT has been traditionally thought to play a minor role in GEP-NENs imaging due to the expected low FDG uptake of low-grade GEP-NENs [136]. As FDG uptake is greater in high-grade tumors, ^18^F-FDG-PET/CT has been proposed in patients with advanced, metastatic GEP-NENs with promising results [137, 138]. In addition, combined functional imaging with both ^68^Ga-DOTATATE and ^18^F-FDG may be useful for a more comprehensive tumor assessment in intermediate and high-grade tumors [125]. Two recent studies confirm that FDG-PET is a sensitive technique for staging GEP-NENs with high (≥10–15 %) Ki-67 [139, 140]. As for other tumors, it has been suggested that FDG positivity points to a worse prognosis [141–143].

^18^F- and ^11^C-labeled amine precursors l-dihydroxyphenylalanine (DOPA) [144–148] and 5-hydroxy-l-tryptophan [146, 149, 150] have been utilized for PET imaging of GEP-NENs in a limited number of studies with promising results. A still investigational tool is ^18^F-fluorothymidine PET that seems to provide non-invasive assessment of cell proliferation. Finally, there is the possibility of utilizing glucagon-like peptide-1 receptor imaging for the localization of insulinomas [151]. Clinical application of these radiopharmaceuticals is not for routine use and needs confirmation.
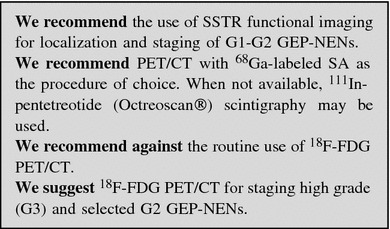


#### Endoscopic procedures

##### Upper and lower gastrointestinal NENs

Upper gastrointestinal endoscopy (EGDS) with gastric biopsy is required for the detection of gastric NENs.

Esophago-gastro-duodenoscopy is the only recommended imaging procedure in small (<1 cm) enterochromaffin-like cell tumors (ECLomas). Type 1 and 2 gastric NENs generally present (in 65–77 % of cases) as small (<2 cm) multifocal polypoid mucosal protrusions in the body and/or fundus of the stomach. Type 3 tumors are usually solitary, ulcerated and larger than 2 cm. In addition to biopsies of the largest polyps, samples should be taken from the antrum (two biopsies) and body/fundus (four biopsies) [152, 153]. Regardless of the type of gastric NEN, EUS may help to determine the presence of tumor invasion of the gastric wall and it is recommended before the resection of polyps >1–2 cm in diameter. EUS is useful for the assessment of the regional lymph node involvement and for cyto-histologic confirmation by FNA [154].

Duodenal NENs are approached in the same manner, namely EGDS with biopsies and EUS [155, 156].

The majority of rectal NENs are diagnosed endoscopically. Most lesions present as polyps, which are completely removed by snare polypectomy, but their diagnosis may be established only after histologic evaluation. Full colonoscopic assessment is required to exclude concomitant colonic disease as part of staging, and the possibility of synchronous carcinoma must be excluded. EUS is very useful in assessing rectal NENs extension preoperatively and it accurately assesses tumor size, depth of invasion and perirectal lymph node metastases. Hence, EUS provides information critical for the choice of final treatment (endoscopic vs. surgical) [157, 158].

##### Small-bowel NENs

Direct visualization of small-bowel NENs may be obtained by standard colonoscopy if the tumor is prolapsed through the ileocecal valve into the colon, or if intubation of the ileum via the ileocecal valve is performed. Newer modalities to investigate the proximal parts of the ileum or the jejunum include video-capsule endoscopy (VCE) and enteroscopy. Small-scale studies have reported successful detection of occult small-bowel NENs by VCE where other techniques have failed. It is advisable to use a dissolvable “patency” capsule to avoid capsule “retention” within strictures. Major VCE limitations are as follows: (a) precise localization of the tumor is not usually possible; (b) in case of predominantly extraluminal GEP-NEN, the evaluation of the tumor cannot be accurate; and (c) cost and operating time. VCE revealed a sensitivity of 60 % and a specificity of 100 % as compared to CT enteroclysis [107, 159].

In selected cases, double balloon enteroscopy (DBE) seems to be a valuable method. It allows histologic confirmation by luminal biopsy and accurate preoperative localization by tumor marking with ink injection. A 33 % diagnostic yield of DBE for primary tumor detection in patients with metastatic or suspected GEP-NEN has been reported [160].

##### Pancreatic NENs

Endoscopic ultrasonography is an effective tool to identify pNENs, which typically appear as well-defined hypoechoic, hypervascular masses. Cystic change, calcifications, and necrosis are common in large tumors. EUS-guided FNA (or biopsy, FNAB) is useful to confirm the diagnosis of pNEN. EUS sensitivity is quite high (79–100 %) with a PPV close to 100 % [161–164]. The accuracy decreases in case of lesions located in the pancreatic tail [165]. While EUS shows a higher sensitivity than cross sectional imaging in the diagnosis of small, multiple pNENs in MEN-1 or VHL syndromes, its accuracy in the detection of small duodenal tumor is controversial. The combination of dual-phase thin-section multidetector CT and EUS has been reported as the most accurate procedure to detect insulinomas [166]. EUS plus FNA is highly cost-effective when used early in the preoperative work-up, reducing the need for additional invasive tests [167, 168]; complication rate is quite low (<1 %) [168]. A close correlation between aspiration cytology and the final histology after resection has been demonstrated [169]. EUS is thus useful in the preoperative setting as it provides information that significantly influences the therapeutic planning [170].
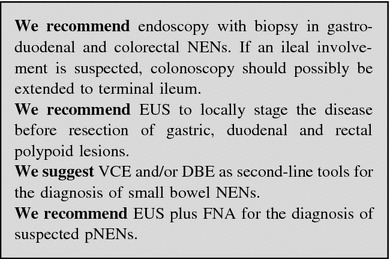


## A step-by-step multidisciplinary approach to clinical diagnosis

The suspicion of GEP-NEN can be raised in four different scenarios: (1) incidental finding either in a totally asymptomatic patient or in a patient with symptoms unrelated to GEP-NEN; (2) symptomatic patient with GEP-NEN-related local effects, (3) syndromes, and (4) metastases from unknown primary GEP-NEN. The first two scenarios are typical of NF GEP-NENs.

### Incidental finding

GEP-NENs are often suspected following incidental imaging (e.g., US, CT, MRI) or endoscopic findings, in patients without signs or symptoms related to GEP-NEN [1, 3, 6].

The patient should be checked for minor GI complains (diarrhea, constipation, peptic disease, gastroesophageal reflux), any palpable mass, skin and metabolic signs/symptoms, possibly suggesting a functioning syndrome. An accurate clinical history of the patient’s family should also be collected to confirm or rule out a hereditary syndrome [6].

#### GEP-NENs suspected at endoscopy

Incidental diagnosis of GEP-NENs often follows the histologic examination of polypoid lesions found during endoscopic procedures in an asymptomatic patient. Otherwise, gastroduodenal and colorectal NENs may be suspected in case of single or multifocal polypoid mucosal protrusions [152, 155, 158], even though no endoscopic finding is highly specific of NEN.

An endoscopic biopsy of the suspected lesion is mandatory. In case, the endoscopic biopsy is either not feasible or non-diagnostic, morphologic imaging studies should be programmed as the second step. Image-guided or laparoscopic biopsy should be discussed by the multidisciplinary team. Functional imaging could subsequently be performed as a complementary staging-prognostic tool.

**Fig. 2:**
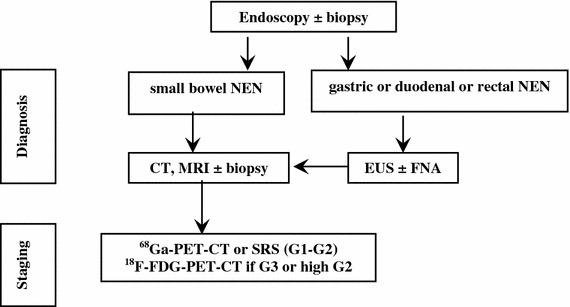
Diagnostic flow-chart for GEP-NEN suspected at endoscopy

No lab tests are indicated in the diagnostic work-up. The finding of hypergastrinemia, achlorhydria, macrocytic anemia, B_12_ deficiency and/or intrinsic factor antibodies may be useful to categorize a gastric NEN (Fig. 2).

#### GEP-NEN suspected at morphological (US/CT/MR) imaging

This incidental finding is usually related to primary pancreatic tumor or liver metastases from a GEP-NEN.

A pNEN might be suspected in case of hypoechoic, hypervascular, and/or well-defined lesions at US/CEUS and of enhancing hypervascular lesions at CT scan or MRI. Cystic changes, calcifications, and necrosis are frequently observed in large lesions [171].

False positives, especially in case of US imaging, like hemangiomas, hepatocellular and pancreatic carcinomas, intraductal pancreatic mucinous tumors, adenomas and metastasis from other tumors [94, 95, 97–101] should be ruled out by the multidisciplinary team.

A histologic/cytological specimen should possibly be obtained [96, 172].

Once the diagnosis of GEP-NEN is pathologically confirmed, proceed to morphologic and functional staging (see below, “When and how to stage a previously diagnosed GEP-NEN”). If biopsy is unfeasible or inconclusive, a second imaging technique (e.g., EUS, CEUS, liver-specific contrast-enhanced MRI, etc.) should be performed according to local expertise and availability [6].

Metastatic lesion(s) from occult primary may require a specific work-up (see below, “Work-up in the patient with metastatic disease and unknown primary tumor”).

**Fig. 3:**
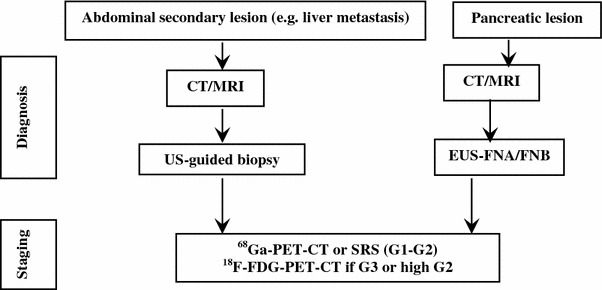
Diagnostic flow-chart for GEP-NEN suspected at morphological imaging

No lab tests are recommended in the diagnostic work-up. Nevertheless, elevated 5-HIAA urinary excretion is highly specific of GEP-NEN liver metastases and may, therefore, be a strong diagnostic clue in case of a non-diagnostic biopsy. In patients with pNENs, the occurrence of subclinical, vague functional signs/symptoms possibly indicating a functional syndrome should always be carefully checked. Accordingly, specific hormonal assays may be required in selected cases (Fig. 3).
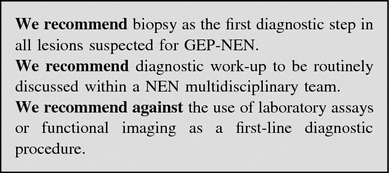

#### GEP-NEN suspected after elevated serum CgA levels

Chromogranin A must never be considered a first-line diagnostic test. Nevertheless, NEN suspicion may occasionally be driven by the finding of elevated serum CgA levels, measured on the basis of unspecific symptoms or signs.

Before proceeding to imaging/endoscopic studies, all factors affecting CgA levels must thoroughly be ruled out (see “Table 3”). A second CgA determination is always required for confirmation. In patients on PPI treatment, serum CgA should be repeated after a two-week PPI withdrawal.

**Fig. 4:**
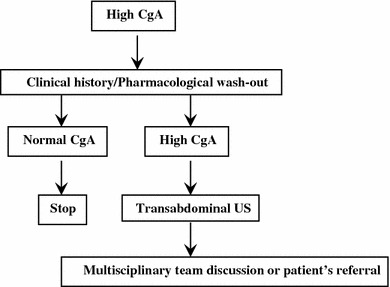
Diagnostic flow-chart for NEN suspected after high CgA

If CgA levels are confirmed elevated in absence of confounding factors, transabdominal US should be performed. A further diagnostic work-up should be discussed by a multidisciplinary team or a referral center should be involved (Fig. 4).
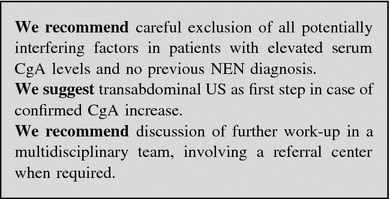

### Symptomatic patient with symptoms due to GEP-NEN-related local effects

#### When to suspect a GEP-NEN

Non-functioning GEP-NENs (Box 1) may become symptomatic when they compress or invade adjacent structures or when they metastasize. The suspicion of GEP-NEN might be raised by suggestive imaging findings (see above) and/or by the apparently slow progression of the disease [73]. Lab findings (e.g., frankly elevated CgA levels in absence of confounding factors) may reinforce the suspicion. As previously stated, only pathology (cytological or histologic characterization), however, will establish the diagnosis [6].

***Box 1*** ***Non-functioning GEP-NENs***

***Definition:*** *NF GEP-NENs are tumors that do not show symptoms related to hormonal hypersecretion. Intracellular hormones or peptides may be demonstrated by IHC, but they are either not secreted, or secreted in quantities unable to elicit a clinical syndrome and/or in an inactive form [* 3 *]. Clinical presentation of NF GEP-NENs depends upon the site of origin and metastases. They can be incidentally discovered when asymptomatic due to the widespread use of diagnostic imaging [* 1 *,* 3 *]. Clinical presentations according to the site of origin are listed below.*

***Pancreas:*** *Up to 60% of pNENs is NF. Most NF pNENs are well differentiated. Annual incidence is 1.8 and 2.6 per million in females and males, respectively [* 3 *]. NF pNEN were traditionally diagnosed late in the course of the disease, with metastases in 46 to 73% of cases, but presently the number of incidentally found small lesions is steeply increasing. Presenting symptoms and signs are abdominal pain (35–78%), weight loss (20–35%), anorexia and nausea (45%), intra-abdominal hemorrhage (4–20%), jaundice (17–50%), and a palpable mass (7–40%) [* 96 *,* 172 *]. NF pNEN may occur in familiar syndromes such as MEN-1, VHL, and TSC.*

***Gastrointestinal:*** *NENs are frequently detected during a screening program or an imaging exam performed to search the primary tumor in an asymptomatic but metastatic patient [* 1 *,* 3 *]. Alternatively, a common clinical presentation is abdominal pain that may be caused by gastro-intestinal dysmotility or obstruction (associated or not to nausea, vomiting or constipation), or by bacterial overgrowth. Less common symptoms and signs are jaundice, weight loss, fatigue, fever and bleeding (massive or dripping). Clinical presentation of appendiceal NEN may mimic acute appendicitis [* 1 *,* 3 *]. Obstructive symptoms are typical of small bowel, whereas minor bleeding is frequent in rectal disease [* 6 *,* 73 *,* 173 *].*

#### Work-up in the patient with local compressive symptoms

A detailed history and complete physical examination are required.

Abdominal pain is the most common presenting symptom of NF GEP-NENs and may be related to the primary tumor or metastatic lesions [1, 3]. Pain localization and characteristics should be carefully examined. Four different scenarios can be distinguished.

##### Isolated abdominal pain

**Fig. 5:**
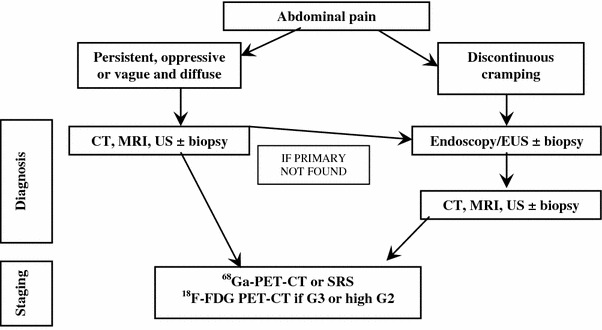
Diagnostic flow-chart for GEP-NEN suspected after pattern 1a and 1b

A persistent and oppressive upper-abdominal pain may signal a pancreatic or retroperitoneal mass (pattern 1a) [96, 172], while a discontinuous cramping pain usually refers to an intestinal origin (pattern 1b) [73, 173]. In the former case, a radiological imaging should be performed first, followed by endoscopy/EUS as second step for pancreatic and duodenal lesions. In the latter case, endoscopy is recommended [73, 173]. A cytologic/histologic sampling should be obtained whenever possible (Fig. 5) [96, 172].

An ill-defined and diffuse abdominal pain (pattern 1c) can also be related to liver or nodal metastases. Abdominal US followed by a whole-body CT scan and a US-guided biopsy should be performed (Fig. 5).

##### Subocclusive picture

**Fig. 6:**
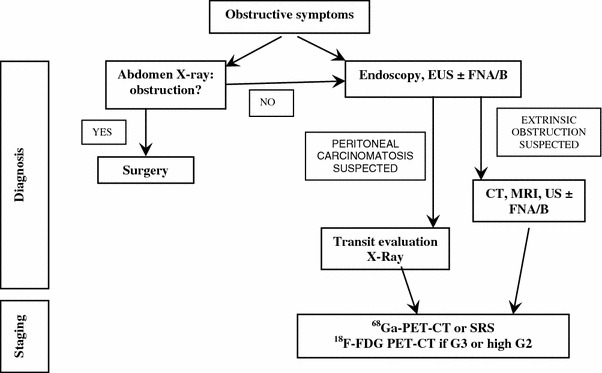
Diagnostic flow-chart for GEP-NEN suspected after subocclusive picture

It may be due to a large, often metastatic, ileal NEN and/or peritoneal carcinomatosis. Depending on the severity of the clinical picture, a direct abdomen-X-ray and/or an endoscopy could be performed [73, 173]. If an extrinsic obstruction is suspected, then an abdomen CT scan should be performed. If a peritoneal carcinomatosis is suspected, a transit evaluation water-soluble contrast medium X-ray could be useful (Fig. 6). If possible, histological specimens should be obtained through endoscopy. If not, a US/CT-guided biopsy of the liver or other site lesions or laparoscopy-guided biopsy should be discussed in a multidisciplinary team.

##### Jaundice

**Fig. 7:**
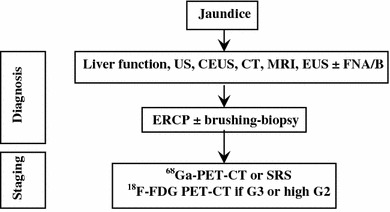
Diagnostic flow-chart for GEP-NEN suspected after jaundice

This clinical presentation points to the involvement of the liver, biliary tract or pancreas. Liver function and structure should be assessed by blood tests and US, to rule out the obstruction of the biliary tract. Compressive effects of lymphadenopathies or a pancreatic mass may cause an extra-hepatic tract dilatation, whereas liver metastases are more likely related to an intra-hepatic tract dilatation [96, 172]. In case of obstructive jaundice, a cholangio-MRI and endoscopic-retrograde-cholangio-pancreatography (ERCP) can be considered. Cytology by means of brushing or histology can be obtained through ERCP. Whole-body CT scan and endoscopy should be used to define the primary site of the tumor and for staging purpose (Fig. 7).

##### Gastrointestinal bleeding

**Fig. 8:**
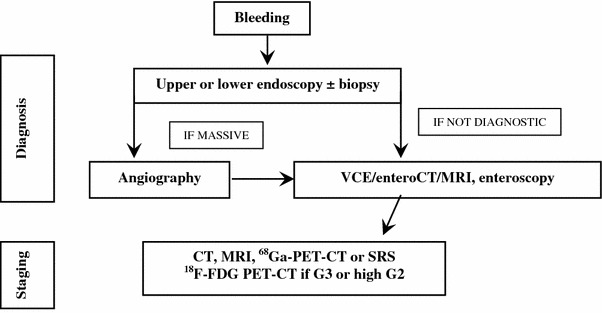
Diagnostic flow-chart for GEP-NEN suspected after GI bleeding

It can be related to the compressive and infiltrating effects of a tumor mass. Bleeding can be massive (hematemesis, melena and rectal bleeding) or dripping and occult. Blood tests, iron assessment and endoscopy must be performed. Massive bleeding always requires hospitalization and may require angiography [73, 173]. In case of lesions located in the stomach-duodenum or in terminal ileum-colon tract, a histologic diagnosis may be obtained through biopsy during EGDS or ileo-colonoscopy. If upper and lower endoscopy is negative, enteroscopy, enteroCT/MRI, VCE should be discussed in the multidisciplinary team according to the local availability and expertise (Fig. 8). For lesions located in the small bowel, a surgical diagnostic/therapeutic approach should be considered
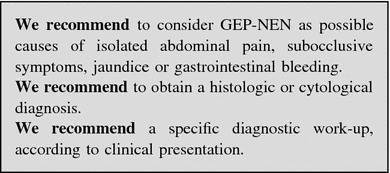

### Symptomatic patient with syndromes

#### Diarrhea and flushing

##### Clinical approach: when to suspect a GEP-NEN

The patient with diarrhea and flushing should raise the suspicion of CS (Box 2).

Carcinoid syndrome diagnosis may be difficult. A detailed history and complete physical examination are must. Symptoms may be under-reported by patients or be attributed to other, more common GI disorders. Differential diagnoses include irritable/inflammatory bowel diseases, microscopic colitis, food intolerance/allergy, bacterial overgrowth, celiac disease, hypersecretory states (i.e., gastrinoma, see “Resistant/relapsing ulcer disease”), chronic pancreatitis, other neoplastic (i.e., colon carcinoma, lymphoma) and non-neoplastic conditions (asthma, anxiety, alcoholism) [6].

*Diarrhea* in patients with CS is chronic, predominantly secretory, does not change with fasting, and is associated with fluid and electrolyte imbalance. A detailed history of the diarrhea and specific questioning about other possible manifestations of CS (i.e., facial flushing) are required. The stools are usually watery and result from intestinal hypermotility and hypersecretion. Nocturnal diarrhea is generally considered as characteristic of CS. The incomplete response to antidiarrhoic treatment should raise the suspicion of possible CS [174].

*Flushing* is the most common symptom in CS. Eating, emotion, alcohol, and exercise may worsen flushing. The face, neck and upper trunk usually turn pink to red in color and the skin is characteristically dry. Flushing may also be associated with transient hypotension and bronchoconstriction. Other causes of flushing/sweating disorders to be considered are [175]:

- pheochromocytoma, menopause, ZES, and medullary thyroid carcinoma (intermittent flushing);
- alcoholism, polycythemia, mitral stenosis, and Cushing’s syndrome (constant flushing).

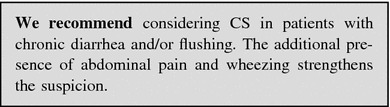

***Box 2*** ***Carcinoid syndrome***

*CS is classified as typical or atypical, accounting for 95% and 5% of total cases, respectively [* 176 *,* 177 *,* 178 *].*

*Typical CS occurs in about 15-20 % of patients with jejuno-ileal NENs, with liver metastases. In less than 5 %, it can be caused by retroperitoneal or ovarian metastases that release serotonin or tachykinin, bypass the liver and enter the systemic circulation [* 179 *,* 180 *,* 181 *,* 182 *]. These so-called “functioning carcinoids” exhibit a variable clinical presentation, due to the type of secreted bioactive substances (serotonin, tachykinins, kallikreins, and prostaglandins). Typical CS may present with cutaneous flushing (face, neck, upper chest), GI hypermotility with pain (intermittent and crampy, described as dull, achy and colicky, and not relieved by defecation), telangiectasia, peripheral edema, wheezing, cyanosis, pellagra, and right-sided heart failure caused by cardiac valve abnormalities. Symptoms may occur spontaneously or be triggered by alcohol intake, serotonin-rich foods, and exercise [* 182 *].*

*Atypical CS is associated to overproduction of histamine and is characterized by prolonged flushing, bronchoconstriction and hypotension [* 178 *]. Wheezing might suggest asthma that can be identified by lung function tests.*

*Carcinoid crisis is an extreme and life-threatening expression of the CS, induced by the massive release of amines into the circulation following anesthesia, interventional procedures or medication [* 183 *]. Main features of carcinoid crisis are: hypotension, rarely hypertension, tachycardia, bronchial wheezing, and central nervous system dysfunction [* 184 *].*

*Carcinoid heart disease affects 10–20 % of the patients at presentation. CS causes a thickening of the heart valves, impairing their proper function, resulting in insufficiency. Heart failure typically involves the right-side valves. Signs and symptoms include fatigue and shortness of breath during physical activity and peripheral edema in 1 out of 5 patients. Up to 50 % of deaths in CS are due to heart failure [* 185 *,* 186 *].*

##### Work-up in the patient with suspected carcinoid syndrome

Before proceeding to the work-up, other causes of flushing with or without diarrhea must be excluded (Table 7) [187]. To this aim it could be useful a 2 to 4-week detailed self-recording of the flushing and diarrhea episodes.

**Table 7:** Differential diagnosis of flushing

Drugs	All vasodilators, calcium channel blockers, morphine and other opiates, etc.
Menopause	Associated with sweating
Mastocytosis	Flushing lasting longer than CS, may be accompanied by headache, dyspnea, palpitations, abdominal pain and diarrhea
Medullary thyroid carcinoma	Associated with diarrhea in patients with advanced disease
Pheochromocytoma	Rare, but it may occur after a paroxysm of hypertension, tachycardia and palpitations and is preceded by pallor

Since symptoms associated with CS can be triggered by alcohol intake and serotonin-rich foods [188–190], the patient should follow an exclusion diet for at least 3 days before starting urinary collection for 5-HIAA and should avoid for at least 24 h (or according to half-life) drugs that affect this test (see Table 4).

Biochemical testing: Urinary excretion of 5-HIAA is the most useful test in patients with typical CS due to jejuno-ileal NENs. Atypical CS is induced by gastroduodenal and bronchial NENs that only rarely secrete serotonin because they lack DOPA-decarboxylase, the enzyme that converts 5-hydroxytryptophan into serotonin [191]. These tumors may thus produce 5-hydroxytryptophan and histamine instead of serotonin, but no assay for urinary 5-hydroxytryptophan is commercially available, whereas histamine assays are limited to very few centers.

5-HIAA testing is highly sensitive (up to 90 %) and specific (85–90 %) for the diagnosis of CS. In patients with CS 5-HIAA levels are usually at least twice as high as the upper normal limit. They may reflect the tumor burden and are rarely normal in patients with CS [57, 73, 76, 77, 192–194]. Attention must always be paid to factors causing falsely high or low levels (see Table 4).

Serum serotonin determination is not recommended because it may vary considerably according to activity and stress levels [73]. CgA is poorly specific whereas NSE has no diagnostic role [77, 193, 195].

Imaging procedures: Carcinoid syndrome is most frequently due to a NEN in the small bowel associated with liver metastases [196]. Therefore liver assessment examinations should be firstly performed, including US/CEUS (useful to drive biopsy), CT and MRI (superior to CT for small lesions) [95, 197–200].

The type of work-up aimed to the detection of the primary tumor (see “Work-up in the patient with metastatic disease and unknown primary tumor”) and to rule out atypical situations should be discussed in a multidisciplinary panel, also taking into account the possible surgical resection.

Functional imaging studies (SRS, or when available ^68^Ga-DOTA-peptide-PET) may help in localizing the primary tumor and small metastases, and as a predictive factor for somatostatin receptor driven therapies. Combination of SRS or PET with CT increases the sensitivity [117].

In case of persistently negative results of morphological and functional studies, the primary tumor may be located by intraoperative palpation [73].

**Fig. 9:**
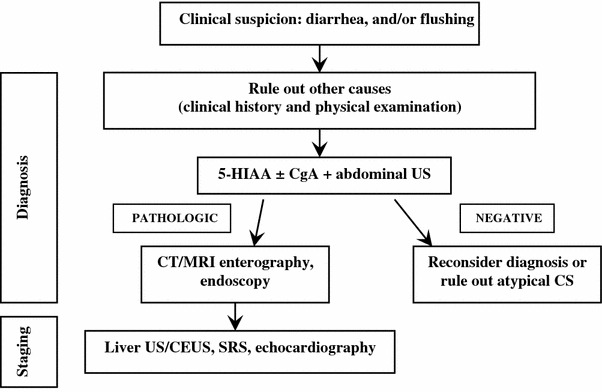
Diagnostic flow-chart for suspected carcinoid syndrome

Transthoracic echocardiography should be performed at diagnosis of CS and then annually to detect any right-sided fibrosis involving tricuspid and pulmonary valves [201] (Fig. 9).
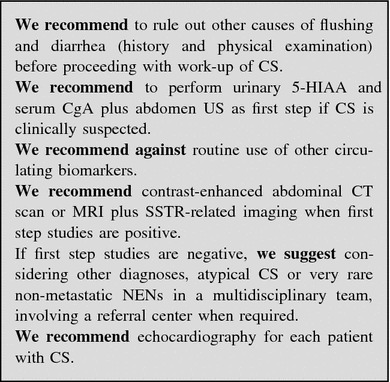
.

#### Resistant/relapsing ulcer disease

##### Clinical approach: when to suspect a GEP-NEN

ZES (Box 3) is characterized by gastric acid hypersecretion resulting in severe peptic disease and gastroesophageal reflux disease (GERD) [202–205].

The majority of ZES patients presents with a single duodenal ulcer, peptic symptoms, GERD symptoms or ulcer complications and diarrhea. Multiple ulcers or ulcers in unusual locations are a less frequent presenting feature than in the past [8, 84, 85, 87, 202, 204–208]. With the widespread use of gastric antisecretory drugs, particularly PPIs and H2RAs, symptoms may be masked. The diagnosis is most often suggested by a long history of peptic ulcer disease or GERD symptoms or their recurrence after treatment [83–85, 206, 208]. This delay may postpone the diagnosis of gastrinoma to a higher stage of the disease.
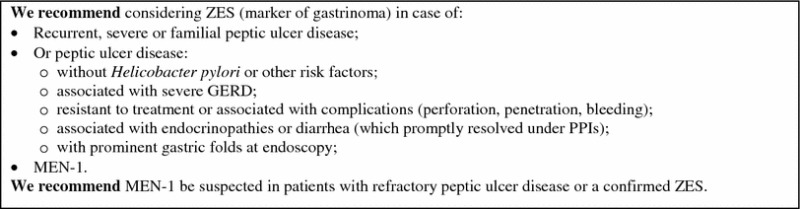

***Box 3*** ***Gastrinoma***

*Gastrinoma is a functioning GEP-NEN, usually located in the duodenum or pancreas that secretes gastrin and causes a clinical syndrome known as ZES.*

*The incidence of gastrinomas is 0.5–2/million population/year. Gastrinoma is one of the most common functioning GEP-NEN in the general population [* 8 *] and occurs in 25–40 % of subjects with MEN-1 [* 207 *,* 209 *]. ZES occurs at an earlier age (mean 32–35 years] in patients with MEN-1 than in those with sporadic disease [* 204 *,* 207 *,* 209 *].*

*Pancreatic gastrinomas may occur in any portion of the pancreas, while duodenal gastrinomas are predominantly found in the first part of the duodenum including the bulb [* 210 *,* 211 *]. At surgery, 70–85 % of gastrinomas are found in the right upper quadrant (duodenal and pancreatic head area), the so-called “gastrinoma triangle” [* 210 *,* 211 *,* 212 *].*

*The main symptoms classically associated to ZES are due to gastric acid hypersecretion and are represented by abdominal pain (75–98 % of the cases), diarrhea (30–73 %), heartburn (44–56 %), bleeding (44–75 %), nausea/vomiting (12–30 %), and weight loss (7–53%) [* 85 *,* 204 *,* 205 *].*

*At presentation, >97 % of patients have an elevated fasting serum gastrin (FSG) level, 87–90 % have marked gastric acid hypersecretion (basal acid output >15 mEq/h) and 100 % have a gastric acid pH <2 [* 202 *,* 213 *].*

*The rate of malignancy is high with liver metastases in 30-40 % of cases [* 214 *].*

##### Work-up in the patient with suspected gastrinoma

History and clinical examination are the first steps in the diagnosis of ZES. The use of acetylsalicylic acid and other non-steroidal anti-inflammatory drugs, which can mimic a ZES picture, should be ruled out [215].

Multiple endocrine neoplasms should be considered in all patients with ZES, especially in case of familial or personal history of endocrine disease, kidney stones, other NENs [88, 207]. Due to high penetrance of primary hyperparathyroidism in MEN-1 [40], serum calcium and PTH are the first step to rule out the diagnosis.

Biochemical testing: Fasting serum gastrin is an excellent screening test (>98 % sensitivity). False positive conditions should always be excluded (Table 5). The diagnosis of ZES requires inappropriately elevated FSG levels in association with a >15 mEq/h (>5 mEq/h in gastrectomized patients) basal acid output or in association with a gastric pH <2.0. Under these conditions, FSG >1,000 pg/mL means a certain diagnosis of ZES. On the contrary, a gastric pH >2.0 virtually excludes ZES [84]. In subjects under chronic therapy with PPIs these drugs have to be withdrawn for at least 1 week [84, 216], although the optimal wash-out time for PPIs should be longer (4 weeks). H2RAs exert a less pronounced suppression of gastric acid output than PPIs [217, 218]. In case of subjects on PPIs who are at risk of bleeding ulcer, diarrhea with dehydration or hypokalemia, these drugs may be replaced with H2RAs for at least 1 week under medical supervision [219, 220].

Secretin test (2 U/kg rapid infusion), a gastrin provocative test, may be performed in controversial cases [77, 221]. Withdrawal of antacid and anticholinergic drugs (12 h), and of PPIs (1 week) is recommended [222]. The secretin test is positive when a >120 pg/mL increase of FSG over the basal value is found (sensitivity 94 %, specificity 100 %) [88, 223]. Calcium stimulation test (5 mg/kg body weight per hour, infused over 3 h, increase >395 pg/mL over the basal FSG as cut-off) may alternatively be used. However, it is hampered by lower sensitivity, specificity and higher side effects [88]. Gastric acid secretion stimuli are no longer performed [203].

Imaging: After biochemical diagnosis, EGDS is required. In ZES, peptic ulcer disease is found distally to the duodenal bulb within the descending part of the duodenum or even further distally within the jejunum. Peptic ulcers frequently occur in groups indicating some substantial acid hypersecretion [84].

The following imaging procedures may be used to localize the primary tumor, determine the extent of the disease, evaluate indication to surgery, and assess response to treatments [88]: (1) contrast-enhanced CT and/or MRI, EUS; (2) functional imaging (SRS, PET); and (3) selective intra-arterial calcium injection angiography [84, 88].

**Fig. 10:**
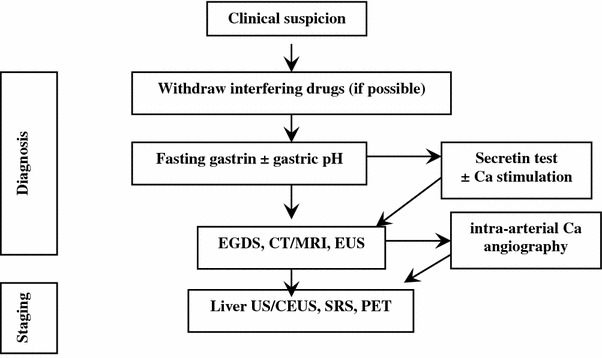
Diagnostic flow-chart for suspected gastrinoma

Accurate localization of the tumor can result in complete surgical resection, decreased rate of developing lymph node metastases, and increasing survival [88, 222, 224–226] (Fig. 10).
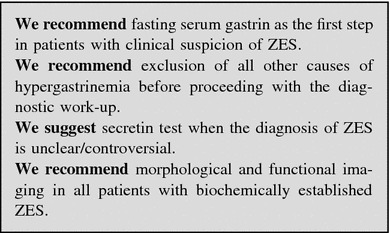

#### Spontaneous hypoglycemia

##### Clinical approach: when to suspect a GEP-NEN

Hypoglycemia (plasma glucose <60 mg/dL on a venous blood sample) is an uncommon clinical problem in non-diabetic adults. The presence of symptoms reinforces the clinical relevance of this finding because some normal subjects may have an asymptomatic low glucose level after prolonged fasting. Symptoms may be due to sympathoadrenal activation (“adrenergic symptoms”, i.e., sweating, shakiness, tachycardia, anxiety, hunger) and/or neuroglycopenia (weakness, dizziness, inappropriate behavior, altered concentration, confusion, blurred vision and, in extreme cases, coma and death) [227–229]. Symptoms may present at a variable glucose level (generally as low as <55–60 mg/dL) [227, 228, 230, 231].

Hypoglycemia may be due to several conditions beyond insulin-secreting tumors [232] (Table 8).

**Table 8:** Differential diagnosis of hypoglycemia

Drugs	Insulin, oral hypoglycemic drugs Quinine, pentamidine, indomethacin, lithium More rarely: ACE-inhibitors, levofloxacin, trimethoprim-sulfamethoxazole, and heparin
Excessive alcohol consumption	Block of stored glucose release
Liver, kidney or heart failure	Depletion of substrates required for gluconeogenesis
Long-term starvation (anorexia nervosa)	Depletion of substrates required for gluconeogenesis
Non-islet cell tumors	Excessive production of IGF-II that causes the use of too much glucose
Gastric surgery (post-gastric bypass)	Accelerated transit and malabsorption
Hypoadrenalism and hypopituitarism	Deficiency of hormones that regulate glucose production
Insulin autoimmune hypoglycemia	

Insulinoma (Box 4) should be strongly suspected in presence of the Whipple triad, which occurs in about 75 % of patients and combines (1) symptoms of hypoglycemia, (2) low blood sugar concurrent with symptoms, and (3) reversal of symptoms after glucose administration [233]. Neuroglycopenic symptoms usually dominate the clinical picture so that insulinoma may be misdiagnosed with cognitive impairment, psychiatric illnesses or seizure disorders. Frequently, the occurrence of bizarre behavior or confusion states is more precisely described by concerned relatives or friends than by the patient himself. Adrenergic and neuroglycopenic symptoms may coexist, especially in the early phase of the disease. A detailed description of pure adrenergic symptoms, however, is more specific of a “functional hypoglycemia”.

Hypoglycemic symptoms occur most frequently at night and/or early morning and, anyway, in a protracted fasting state. Yet, the occurrence of post-prandial hypoglycemia does not exclude an insulinoma [234, 235]. Symptoms can be worsened by exercise, alcohol, hypocaloric diet, and by concomitant clinical conditions or use of drugs (see above) [236, 237]. Weight gain occurs in 20–40 % of patients that may develop overweight because of hyperinsulinism.
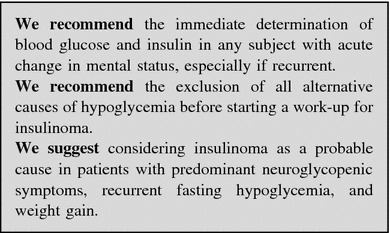

***Box 4*** ***Insulinoma*** ***[*** 236 ***,*** 237 ***]***

*Insulinoma is a NEN arising from insulin-secreting cells in pancreatic islets. Other hormones and metabolites (gastrin, ACTH, glucagon, hCG, somatostatin, and 5-HIAA) may be also secreted from this neoplasm.*

*About 90 % of insulinomas are benign. In rare cases neither a single nor multiple tumors can be identified and the syndrome depends on diffuse beta-cell hyperplasia. In malignant forms with liver metastases, a 16–26 months survival is to be expected. Only 5 % of all insulinomas are associated with MEN-1; in case of multiple insulinomas (near 10%), MEN-1 prevalence raises to 50 %.*

##### Work-up in the patient with suspected insulinoma

Biochemical assessment: Symptoms and/or signs suggesting hypoglycemia combined with a ≤55 mg/dL (3.0 mmol/L) plasma glucose, a ≥3.0 μU/mL (18 pmol/L) plasma insulin, a ≥0.6 ng/mL (0.2 nmol/L) C-peptide, and a ≥5.0 pmol/L proinsulin indicate endogenous hyperinsulinism [227, 228, 231]. Exogenous insulin-induced hypoglycemia is always associated with low levels of C-peptide. In patients with insulinoma, proinsulin corresponds to about 70 % of insulin immunoreactivity, whereas it is normally limited to 20 %.

Blood and urine assays for sulfonylureas will detect factitious hypoglycemia caused by these drugs. Pituitary and adrenal function tests are useful to rule out hypoadrenalism and hypopituitarism [227, 228, 230, 238].

Provocative tests: Biochemical diagnosis is based on lack of suppression of endogenous insulin secretion by hypoglycemia [239] and inappropriately elevated insulin level during hypoglycemia is the diagnostic key point. In 95 % of cases, the diagnosis is achieved only during prolonged fasting (up to 72 h) inducing symptomatic hypoglycemia [240]. The test should be performed on inpatients under close supervision and with regular control of glycaemia and mental status. A ≥3 µU/mL (≥18 pmol/L) insulin value, in the presence of glucose level <55 mg/dL has recently been proposed as diagnostic cut-off [223]. Plasma β-hydroxybutyrate levels ≤2.7 mmol/L may confirm the diagnosis, demonstrating the suppressive effect of insulin on ketogenesis even during a protracted fasting [231].

At the end of the 72-h fasting test, in the absence of hypoglycemia, the use of stimulation tests was proposed [231]. Stimulation tests, e.g., tolbutamide, glucagon or calcium, are not recommended because they may induce a prolonged and refractory hypoglycemic condition, but long-term fasting can be finished after 72 h with bicycle test.

Imaging: In all patients with a confirmed biochemical diagnosis, imaging is indicated to localize the tumor [241].

Since 80 % of insulinomas are <2 cm in size, they are frequently missed by high-resolution transabdominal US (50 % sensitivity), while EUS is more sensitive (77 %) and should be preferred [242]. Helical or multislice CT and MRI offer a comparable (82–94 %), but incomplete, sensitivity [243, 244]. Selective arteriography has an 82 % sensitivity and a 95 % specificity.

Due to small size and/or lack of SSTR2 expression in 50 % of insulinoma [151], SSTR-related imaging plays a minor role than morphological imaging. DOPA-PET has been proposed as an alternative tool [245]. Radiolabelling with ^111^In-labeled glucagon-like peptide-1 receptors agonist (^111^In-DOTA-exendin-4) is a promising technique, still not routinely used [246].

Arteriography combined with selective calcium stimulation: Calcium is able to stimulate insulin release from neoplastic tissue, but not from normal islets. Hence, the catheterization of the arterial branches of the celiac system and the measurement of insulin in the blood sampled from hepatic veins during selective intra-arterial calcium injection localize the pancreatic area nesting the tumors in 88–100 % of cases [34, 247, 248]. This test is cumbersome, expensive and poorly available. Accordingly, it should be reserved only to selected, biochemically proved cases with negative imaging studies.

**Fig. 11:**
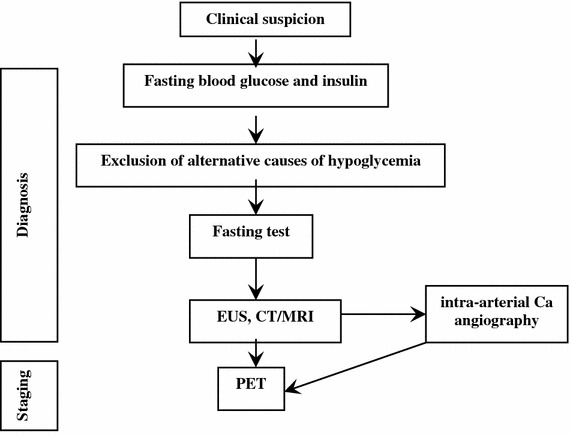
Diagnostic flow-chart for suspected insulinoma

In spite of all the above reported diagnostic techniques, only 60–70 % of patients have a successful preoperative localization. In patients with less threatening symptoms that are fairly controlled by medical treatment a close surveillance may be advisable. In severely symptomatic cases, the use of intraoperative US and the pancreatic exploration conducted by an experienced surgeon identifies more than 90 % of the insulin-secreting tumors [242, 249] (Fig. 11).
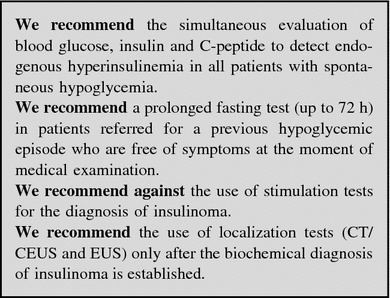

### Work-up in the patient with metastatic disease and unknown primary tumor

Unknown primary NEN (UPN) is a condition of metastatic histologic or cytological confirmed NEN without evidence of a primary site after a first diagnostic work-up, including chest-abdomen CT scan, SRS, and upper and lower endoscopy.

The frequency of well-differentiated UPNs ranges from 9 to 19 % [250, 251]. The presence of liver metastases largely influences prognosis in all types of NENs and is dependent on primary tumor site, tumor extent (T-stage), and histologic differentiation (NET vs. NEC). Reported survival rate at 5 years of G1–G2 small intestinal and pancreatic NENs in the SEER database is 54 and 27 %, respectively [252]. Furthermore, survival is reportedly worse in UPN patients as compared to patients with liver metastases whose primary NEN is known [253]. In liver metastatic patients survival rate is influenced by the presence of obstructive symptoms or symptoms related to peptide secretion.

The evaluation of a patient with UPN should encompass a detailed clinical history, including family history to identify affected relatives and a patient’s increased risk for endocrine tumors (i.e., MEN type 1 or 2), laboratory and radiographic studies [254].

Histologic preparations should be reevaluated by IHC to guide the search for the primary tumor: TTF-1 (pulmonary or medullary thyroid carcinoma), CDX-2 (intestinal), PAX-8, histidine-decarboxylase (pancreatic), xenin (duodenal), gastrin (occult gastrinoma), and PP/glucagon (pancreatic) [38, 255]. Biochemical work-up may include 5-HIAA, gastrin, and other locally available tumor markers [256].

**Fig. 12:**
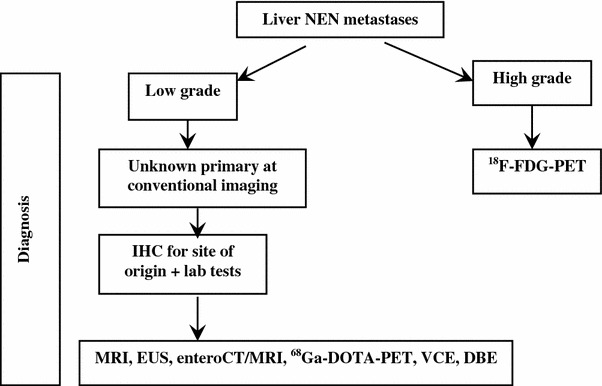
Diagnostic flow-chart in the patient with metastatic disease and unknown primary tumor

It has been recently reported that most UPNs are derived from pancreas and small bowel [257]. Accordingly, further investigations for localizing the primary site in well-differentiated NENs might include abdomen MRI, EUS, enteroCT/MR, ^68^Ga-PET, VCE, DBE to be shared within a multidisciplinary team according to clinics, local availability and expertise [124, 258, 259]. In NECs ^18^F-FDG-PET may be useful (Fig. 12)
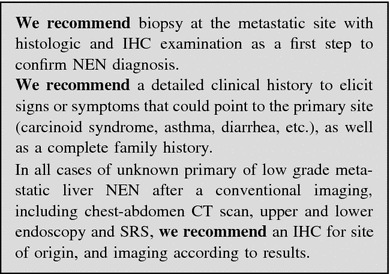

### When and how to stage a previously diagnosed GEP-NEN

Evaluation of disease extension has a pivotal role in treatment planning.

Pre-treatment staging should include morphologic and functional imaging. Morphological imaging is required for all GEP-NENs, irrespectively of their grade. SSTR-based functional imaging (SRS or ^68^Ga-DOTA-peptide-PET) should be used for low-/intermediate-grade GEP-NENs (WHO 2010 G1-G2), whereas ^18^F-FDG-PET should be preferentially used in G3 GEP-NENs and in some G2 cases.

For morphologic staging, a chest-abdomen-pelvis multidetector CT or a chest basal CT plus abdomen-pelvis MRI should be used [87]. For functional staging, SRS using ^111^In-pentetreotide (Octreoscan^®^) is presently regarded as the gold standard. However, if available, ^68^Ga-DOTA-peptide-PET with simultaneous CT should be preferred to SRS. In facts, PET lacks the anatomic details required for therapeutic stratification (surgical planning or dose calculation for radioembolization with radiolabeled microspheres). Recently, MRI with liver-specific contrast combined with ^68^Ga-DOTA-peptides-PET has been reported to be more accurate than PET-CT to detect GEP-NEN hepatic metastases [260].

^18^F-DOPA-PET-CT and ^11^C-5HTP-PET-CT are promising tools. Their use might be considered if results of SRS or ^68^Ga-DOTA-peptides-PET are negative [261].

*Gastric NENs* In small (<1 cm) type 1 and type 2 tumors, EGDS is usually the only recommended imaging procedure [153]. Tumor invasiveness through the gastric wall must be evaluated with EUS study: it is recommended before resection for polyps >1 cm in diameter. EUS is also useful in assessing regional lymph nodes involvement, and allows histological confirmation by FNA. Type 1 tumors do not require either abdomen multislice CT or MRI, or SRS/^68^Ga-DOTA-peptides-PET; these imaging studies should be performed for type 2 and type 3 neoplasm staging.

*Duodenal NENs* EUS is useful before resection of polypoid lesions; multislice abdomen CT or MRI should be performed to assess local and distant disease extension. In patients with local advanced neoplasm and/or liver metastases, bone scan and MRI of spine and pelvis should be performed [153].

*Jejuno-ileum NENs* Chest-abdomen-pelvis CT scan or chest basal CT scan and abdomen-pelvis MRI, SRS or ^68^Ga-DOTA-peptides-PET should be performed looking for distant metastases [73]. Liver CT scan should be performed by multislice and multiphase technique. Colonoscopy should be performed to rule out synchronous colorectal carcinoma.

*Colorectal NENs* Chest-abdomen-pelvis multislice CT should be carried out. Endoanal/rectal US is very useful for assessing preoperatively the depth of tumor invasion in the rectal wall and regional lymph node involvement [173].

*NF pNENs* For morphologic staging a multislice/multiphase CT or fat-saturated T1-weighted and delayed enhanced T1-weighted MRI can be performed and EUS with biopsy [262]. Afterwards, SRS or ^68^Ga-DOTA-peptides-PET should be performed.
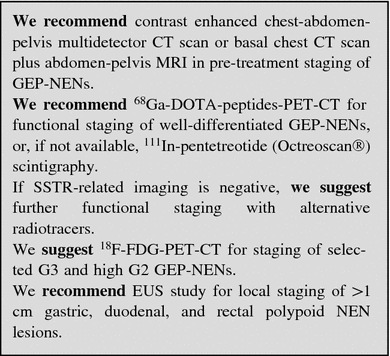


## Conclusions

The management of patients with GEP-NENs poses a significant challenge to clinicians from the very start of the diagnostic work-up. The wide heterogeneity of disease presentation, with a majority of asymptomatic patients and poorly specific clinical pictures may account for a delay in definite diagnosis and appropriate treatment. The present document has, therefore, been drawn with the purpose of offering a practical guide to physicians facing the suspicion of GEP-NENs, in light of the available clinical evidence and experience. Of course, many questions are still to be fully answered and many others still to be addressed in the near future, as we move forward to new promising techniques and diagnostic tools. For these reasons, in spite of its goal as a state-of-the-art update, our document has not been conceived as the repository of the “ultimate truth” in the field of GEP-NENs diagnosis. Instead, much attention has been devoted to the logical framework, which should back up the clinical reasoning. Furthermore, the diagnosis of GEP-NENs is heavily based on the contribution of a wide range of know-how and skills provided by different specialists. The core team may include a varying combination of different specialists, according to the local expertise and facilities; nevertheless, the pathologist plays a key role in the diagnosis and classification of GEP-NENs, because his/her information is critical to guide the prognosis and treatment planning. Hence, a multidisciplinary team model is recommended as the best opportunity to reach an accurate, safe and cost-effective diagnosis, likely to improve the outcome of patients with GEP-NENs.

In conclusion, the Italian Association of Clinical Endocrinologists (AME) hopes the present Position Statement will constitute an effective tool in helping the clinical management of patients with GEP-NENs. Further implementations and updates of this document will follow as new evidence and progress in the field emerge.

*Other members of AME oncologic endocrinology group* Giorgio Borretta, Cuneo; Renato Cozzi, Milan; Giuseppe Francia, Verona; Rinaldo Guglielmi, Albano Laziale; Gabriele Luppi, Modena; Salvatore Monti, Rome; Silvia Nasoni, Albano Laziale; Micaela Pellegrino, Cuneo; Anna Pia, Turin; Sara Pusceddu, Milan; Valeria Ramundo, Naples; Francesco Scavuzzo, Naples; Alessandro Scoppola, Rome; Ettore Seregni, Milan; Francesca Spada, Milan; Laura Tonutti, Udine; Vincenzo Toscano, Rome; Maria Chiara Zatelli, Ferrara.

## Abbreviations

5-HIAA: 5-Hydroxy-indolacetic acid
ACE: Angiotensin-converting enzyme
ACTH: Adrenocorticotropin
AJCC: American Joint Committee on Cancer
AKT: A protein-serine-threonine kinase that is activated by phosphorylation in response to growth factors or insulin
CD117: Antigen specific for the proto-oncogene *c*-*kit*
CD56: Antigen expressed by all lymphocytes
CD99: Cluster of differentiation
CDX-2: Transcription factor expressed specifically in gut epithelium
CEACAM1: Cell adhesion molecule
CEUS: Contrast-enhanced US
CgA: Chromogranin A
CK19: Cytokeratin 19
CS: Carcinoid syndrome
CT: Computerized tomography
DBE: Double balloon enteroscopy
DOPA: Dihydroxyphenylalanine
DOTA: 1,4,7,10-Tetra-azacyclo-dodecane-tetraacetic acid
DOTANOC: DOTA-Nal3-octreotide
DOTATATE: DOTA-octreotate
DOTATOC: DOTA-edotreotide
ECLomas: Enterochromaffin-like cell carcinoids
EGDS: Esophago-gastro-duodenoscopy
ELISA: Enzyme-linked immunosorbent assay
ENETS: European Neuroendocrine Tumor Society
ERCC-1: Excision repair cross-complementing
ERCP: Endoscopic-retrograde-cholangio-pancreatography
EUS: Endoscopic ultrasonography
FDG: Fluoro-deoxy-glucose
FGF: Fibroblast growth factor
FNA: Fine needle aspiration
FNB: Fine needle biopsy
FSG: Fasting serum gastrin
GEP: Gastroenteropancreatic
GERD: Gastroesophageal reflux disease
GI: Gastrointestinal
GRADE: Grading of recommendations, assessment, development, and evaluation
hCG: Human chorionic gonadotropin
hHAS-1: Human achaete-scute homolog 1
H2RAs: Histamine H2-receptor antagonists
Her/2: A cell surface protein-tyrosine kinase receptor overexpressed in adenocarcinomas
HPF: High-power field
HPLC: High-pressure liquid chromatography
HTP: Hydroxy-l-tryptophan
IHC: Immunohistochemistry
IRMA: Immunoradiometric assay
Ki-67: Nuclear antigen present only in the nuclei of cycling cells
LoE: Level of evidence
MANEC: Mixed adeno-neuroendocrine carcinoma
MAO: Monoamine oxidase
MDCT: Multidetector CT
MEN-1: Multiple endocrine neoplasms
MGMT: Methylguanine-DNA methyltransferase
MIB-1: A monoclonal antibody used to detect KI-67 antigen
MRI: Magnetic resonance imaging
mTOR: Mammalian target of rapamycin
NEC: Neuroendocrine carcinoma
NEN: Neuroendocrine neoplasm
NET: Neuroendocrine tumor
NF: Non-functioning
NF1: Neurofibromatosis type 1
NIH: National Institutes of Health
NPV: Negative predictive value
NSE: Neuron-specific enolase
NSP-55: Neuroendocrine-specific protein
OS: Overall survival
PAX-8: Paired box gene 8
PET: Positron emission tomography
PGP.9.5: Pan neuronal marker protein in the Islets of Langerhans
PIK3: Phosphoinositide-3-kinase
PLGF: Placental growth factor
pNEN: Pancreatic NEN
PPIs: Proton pump inhibitors
PPV: Positive predictive value
PTEN: Phosphatase and tensin homolog (mutated in multiple advanced cancers)
RIA: Radioimmunoassay
SA: Somatostatin analog
SEER: Surveillance, epidemiology and end results
SPECT: Single photon emission computed tomography
SRS: Somatostatin receptor scintigraphy
SSTR: Somatostatin subtype receptor
TNM: Tumor-node-metastases
TSC: Tuberous sclerosis complex
TTF-1: Thyroid transcription factor 1
UICC: Union for International Cancer Control
UPN: Unknown primary NEN
US: Ultrasonography
VCE: Video-capsule endoscopy
VHL: von Hippel-Lindau disease
VIP: Vasoactive intestinal peptide
ZES: Zollinger–Ellison syndrome
